# Lipopolysaccharide-Induced Vascular Inflammation Model on Microfluidic Chip

**DOI:** 10.3390/mi11080747

**Published:** 2020-07-31

**Authors:** Ungsig Nam, Seunggyu Kim, Joonha Park, Jessie S. Jeon

**Affiliations:** 1 Department of Mechanical Engineering, Korea Advanced Institute of Science and Technology, Daejeon 34141S, Korea; usnam@kaist.ac.kr (U.N.); ksg5825@kaist.ac.kr (S.K.); joonha0306@kaist.ac.kr (J.P.); 2 KAIST Institute for Health Science and Technology, Korea Advanced Institute of Science and Technology, Daejeon 34141, Korea

**Keywords:** microfluidic device, inflammation, lipopolysaccharide, endothelial cell, THP-1 cell

## Abstract

Inflammation is the initiation of defense of our body against harmful stimuli. Lipopolysaccharide (LPS), originating from outer membrane of Gram-negative bacteria, causes inflammation in the animal’s body and can develop several diseases. In order to study the inflammatory response to LPS of blood vessels in vitro, 2D models have been mainly used previously. In this study, a microfluidic device was used to investigate independent inflammatory response of endothelial cells by LPS and interaction of inflamed blood vessel with monocytic THP-1 cells. Firstly, the diffusion of LPS across the collagen gel into blood vessel was simulated using COMSOL. Then, inflammatory response to LPS in engineered blood vessel was confirmed by the expression of Intercellular Adhesion Molecule 1 (ICAM-1) and VE-cadherin of blood vessel, and THP-1 cell adhesion and migration assay. Upregulation of ICAM-1 and downregulation of VE-cadherin in an LPS-treated condition was observed compared to normal condition. In the THP-1 cell adhesion and migration assay, the number of adhered and trans-endothelial migrated THP-1 cells were not different between conditions. However, migration distance of THP-1 was longer in the LPS treatment condition. In conclusion, we recapitulated the inflammatory response of blood vessels and the interaction of THP-1 cells with blood vessels due to the diffusion of LPS.

## 1. Introduction

Inflammation, the response to harmful stimuli, is the initiation of the defense mechanism of our body [1]. Inflammation is induced by external stimuli such as molecules containing pathogen-associated molecule pattern (PAMP) [2] from bacterial and viral infection but also molecules produced in our body such as oxidized low density lipoprotein [3]. The inflammatory response of blood vessels, or endothelial cells is important in pathogenesis of diseases [4]. In addition, dysfunction in response to inflammation of blood vessels leads to excessive or non-resolving inflammation, which could be cause of diseases such as atherosclerosis [5], sepsis [6], and vasculitis [7]. Lipopolysaccharide (LPS), which is a component of the outer membrane of Gram-negative bacteria, induces an inflammatory response in the animal’s body; therefore, this is extensively used in models for studying inflammation [8]. Moreover, LPS is not only associated with several diseases such as liver damage, neurological degeneration, chronic inflammation of the gut, and diabetes [9], but also initiates inflammatory response and injury of endothelial lining of blood vessels, or endothelial cells [10]. Therefore, it is important to understand how blood vessels respond to LPS.

The inflammatory response to LPS of blood vessel has been widely studied in vitro. Several recent studies have treated LPS into monolayer of endothelial cells, then tested the anti-inflammation effect of target materials [11,12,13,14]. Other studies showed dose- or time-dependent LPS exposure of endothelial cells to optimize inflammation of their research [4,15,16,17,18,19,20,21,22,23]. These researches used two dimensional (2D) monolayer of endothelial cells that differ from in vivo environment in terms of absence of extracellular matrix (ECM) and dynamic distributions of oxygen, nutrients and other molecules produced by cells [24,25]. On the other hand, microfluidic chips, which have been actively researched, can construct tissue in three dimensions (3D) using extracellular matrix (ECM) and human-derived cells, and are easy to control flow (shear stress) in physiological levels and chemical distributions [26]. With these advantages, a number of previous works which studied the inflammatory response of LPS on blood vessels used microfluidic system [27,28,29,30]. For example, Du et al. [28] established microenvironment of liver in microfluidic chip by co-culturing four types of murine hepatic cells and applying shear flow and found that increase of neutrophil adhesion as the number of co-cultured cell increase when treating LPS. Gröger et al. [29] developed a liver model composed of hepatocyte layer and endothelial cell layer in microfluidic chip, induced inflammation-treating toll-like receptor agonists including LPS, and observed monocyte-induced recovery of inflammation. Jain et al. [30] microfluidic lung alveolus-on-a-chip composed of alveolar epithelium layer and endothelial cell layer. They revealed that LPS indirectly stimulates intravascular thrombosis by activating the alveolar epithelium and analyzed inhibition of endothelial activation and thrombosis due to a protease activated receptor-1 (PAR-1) antagonist. However, there is still a need for the systematic studies on the response of blood vessels with the exposure time of the LPS and resulting response of immune cells still remains.

In this paper, we focused on the inflammatory response of blood vessels on microfluidic chips infiltrated by LPS from the basal side of blood vessels to mimic a situation where blood vessels are invaded by source of LPS such as bacteria. To achieve this, the LPS exposure of a blood vessel constructed in the microfluidic device was designed to be increased with time. By combining simulation and experimental analysis, we show the severity of inflammation of the blood vessel with the exposure time of the LPS of which concentration infiltrating blood vessels increased with time. Furthermore, we conduct image-based analysis of Intercellular adhesion molecule-1 (ICAM-1) and VE-cadherin expression on endothelial cells of constructed blood vessels when inflammation is induced. Finally, we used monocytic THP-1 cells that have been used as immune cell model in numerous studies [31] to mimic the immune response of monocyte including adhesion and trans-endothelial migration behaviors which are important acute inflammatory processes [32] in inflamed blood vessels.

## 2. Materials and Methods

### 2.1. Microfluidic Device Fabrication

**Figure 1:**
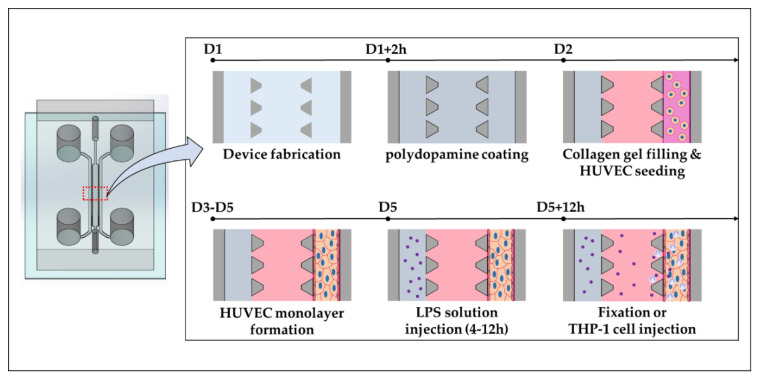
Schematic diagram of fabrication process for recreating blood vessel inflammation model in the microfluidic chip.

The microfluidic device design consists of two side media or cell channels adjacent to the inner gel channel and was utilized previously in the lab (Figure S1A) [33]. The device fabrication is well explained in previous work [34]. Briefly, the master mold was fabricated using photolithography. SYLGARD 184 silicone elastomer base and the curing agent (DOW corning, Midland, MI, USA) was mixed with 10:1 (*w*/*w*) ratio. The bubbles of the mixture were removed and the mixture was cured in a 75 °C oven. PDMS was cut and punch to make reservoirs and inlet of hydrogel. PDMS and cover glasses were sterilized by 70% ethanol, then assembled by plasma treatment. The assembled microfluidic devices were coated with 2.0 mg/mL polydopamine for enhanced adhesion of the hydrogel to the device (Figure 1) [35]. After washing channels of microfluidic device with distilled water, the device was incubated in a 37 °C incubator for more than 12 h.

### 2.2. Cell Culture

Human umbilical vein endothelial cells (HUVEC, ATCC, Manassas, VA, USA) were cultured with Microvascular Endothelial Cell Growth Medium-2 BulletKit (EGM-2MV, Lonza, Basel, Switzerlands) supplemented with 1% (*v*/*v*) Antibiotic-Antimycotic (100×) (Gibco, Gaithersburg, MD, USA) in an incubator (37 °C, 5% CO_2_). Monocytic THP-1 cells (Korean cell line bank, Seoul, Korea) were cultured with RPMI 1640 medium (Gibco, USA) supplemented with 10% (*v*/*v*) heat-inactivated fetal bovine serum and 1% (*v*/*v*) Antibiotic-Antimycotic (100×).

### 2.3. Human Umbilical Vein Endothelial Cells (HUVEC) Monolayer Culture in Microfluidic Device

The blood vessel was constructed by forming 3D HUVEC monolayer in microfluidic device (Figure 1), which was reported in the previous work [34]. Briefly, 2.0 mg/mL and pH 7.0–7.4 collagen gel solution was prepared by mixing the rat-tail collagen type 1 solution (Corning, Corning, NY, USA), 10× phosphate buffered saline (PBS, Lonza, Basel, Switzerland), distilled water, and sodium hydroxide (NaOH, 0.5 N). The gel channel was filled with collagen gel solution and incubated in an incubator for more than 30 min for gelation. One of the media channels (Blood vessel channel, BV channel, hereafter) was coated with 1.0 mg/mL matrigel (Corning, USA), and then washed with Endothelial Cell Growth Basal Medium-2 (EBM-2, Lonza) [36]. The other media channel is filled with EBM-2. When HUVECs on culture dish were confluent, the HUVECs were trypsinized by Trypsin-EDTA (0.25% Trypsin, 1×). The suspension of 3.0 × 10^6^ HUVECs /mL were prepared and 30 μL of the HUVEC suspension was seeded in the BV channel. In order to form HUVEC monolayer and minimize inflammation and angiogenesis, reduced EGM-2MV (rEGM) was used. rEGM was made by excluding VEGF, FGF and EGF from EGM-2MV which are pro-inflammatory features [37]. An hour after seeding, each reservoir of the BV channel was filled with 50 μL of rEGM and medium in the other reservoirs was replaced with 50 μL of EBM-2. Finally, the medium of each reservoir is replaced daily until HUVEC monolayer was formed in the channel (2–3 days).

### 2.4. Simulation of Lipopolysaccharide (LPS) Diffusion

Computational simulation of LPS diffusion was conducted using COMSOL Multiphysics 5.3. Physic model of Transport of Diluted Species in Porous Media which employs Darcy’s law and Brinkman Equation [38] was used to simulate LPS diffusion in collagen gel. The porosity of 2.0 mg/mL collagen gel was defined by 0.8 [39] and diffusion coefficient of LPS was set to 3.0 × 10^−11^ m^2^/s which is between values measured in previous study [40]. Since the molecular weight of LPS provided by the supplier (Sigma-aldrich, St. Louis, MO, USA) is 50–100 kDa, concentration of initial LPS was defined by 1.4286 mol/m^3^ converted from 10 μg/mL assuming molecular weight is 70 kDa. To mimic the endothelial barrier, a thin diffusion barrier was set as layer thickness set by 2 μm measured by confocal image. Diffusion coefficient of endothelial barrier was set to 7.4 × 10^−14^ m^2^/s, which is calculated by the following equation as reported previous study [41]:

$$P=\frac{KD}{x_{0}}$$

In this equation, *P* is permeability, *D* is diffusion coefficient, *x*_0_ is thickness of cell, and *K* is partition coefficient. Since only para-cellular diffusion was considered, solvent of LPS was not changed, resulting *K* = 1. Thus, equation above could be simplified into *D* = *Px*_0_ where *x*_0_ is 2 μm and *P* is 3.70 × 10^−8^ m/s from previous research [42].

### 2.5. LPS Treatment and THP-1 Cell Adhesion/Migration Assay

Lipopolysaccharide solution of 500 μg/mL is made by solving LPS from *E. coli* (O55:B5) (Sigma Aldrich) in PBS. The LPS solution was further diluted with EBM-2 to final concentration of 10 μg/mL, for which the concentration is enough to observe gradually increasing LPS in the constructed blood vessel. When the HUVEC monolayer was formed, covering the entire surface of microchannel, the LPS solution (10 μg/mL) was injected into channel opposite to the BV channel to stimulate inflammation. For inflammatory condition, microfluidic devices were incubated for 4, 8, 12 h with the LPS solution. For normal conditions (control), media in channel opposite to the HUVEC monolayer were replaced with 2 μL of PBS is mixed with 98 μL of EBM-2 to minimize the effect of PBS. After the duration of the incubation, the samples were fixed for immunofluorescence staining or further used as THP-1 cell adhesion/migration assay without fixation. In THP-1 cell adhesion/migration assay, THP-1 cell suspension was prepared at 3.0 × 10^5^ THP-1 cells/mL in rEGM, and 25 μL of THP-1 cell suspension was injected into the BV channel three times. Each injection was conducted when flow of THP-1 cell suspension in the BV channel stopped (about 5 min interval). The samples were fixed after 6 h incubation from the final THP-1 cell injection, because it was enough for THP-1 cells to adhere to endothelium and extravasate while refraining the collagen gel from degrading which occurred when the incubation was longer than 6 h.

### 2.6. Staining and Image Analysis

Before THP-1 cell suspension was injected, THP-1 cells were tagged with CellTracker Green CMFDA Dye (Invitrogen, Carlsbad, CA, USA). The solution was prepared by a 10-μM CellTracker Green CMFDA Dye solution in serum free RPMI 1640 medium. The samples were fixed with 4% paraformaldehyde and permeabilized with 0.2% triton-X 100 solution. The nucleus and actin filament of the samples were stained with 4′, 6-diamidino-2-phenylindole (DAPI) and Rhodamine Phalloidin, respectively. The samples were blocked using CAS-Block Histochemical Reagent (Thermo Fisher, Waltham, MA, USA). For the primary antibody, ICAM-1 Monoclonal Antibody (Invitrogen, Carlsbad, CA, USA) diluted to 1:100 and Anti-VE Cadherin antibody—Intercellular Junction Marker (abcam) diluted to 1 μg/mL was used. For secondary antibody, Goat Anti-Mouse IgG H&L (Alexa Fluor^®^ 488) (Abcam, Cambridge, England) diluted to 1:1000 was used to conjugate ICAM-1 primary antibody. Goat anti-Rabbit IgG (H + L) Highly Cross-Adsorbed Secondary Antibody, Alexa Fluor 488 (Invitrogen) was diluted to 1:100 used to conjugate VE-cadherin primary antibody. Dummy samples were stained with same concentration of respective secondary antibody without primary antibody.

The confocal fluorescent images of ICAM-1 and VE-cadherin were obtained using LSM 880 (Carl Zeiss, Oberkochen, Germany) with detection wavelength 490–552 nm for Alexa 488, 410–494 nm for DAPI, and 566–695 nm for Rhodamine. The fluorescent images of THP-1 adhesion/migration assay were obtained using Axio Z1 (Carl Zeiss). Three images per a device were acquired in the vascular channels at region of interest for ICAM-1 and VE-cadherin (Figure S1B). On the other hand, THP-1 adhesion/migration assay images were acquired in overall BV channels. The obtained images were processed using ZEN 2.6 (blue edition). In order to analyze data from images, firstly, the stacked images were processed by maximum intensity projection for nucleus, ICAM-1, and VE-cadherin. Images for actin filaments were generated by either maximum or average intensity projection, because some signal of actin filaments were too intense to cause image bleeding. In addition, images for cells on ECM side were made from stacking images by average intensity projection method for nucleus, actin filaments, and ICAM-1 and VE-cadherin. Since below the top 1% of fluorescent intensity of the dummy samples was considered as noise, intensity value 0~9 from an 8-bit Alexa 488 channel in ICAM-1 images and intensity value 0–14 from the 8-bit Alexa 488 channel in VE-cadherin images were excluded from analysis. The area and fluorescent intensity of the processed images was analyzed by ImageJ (NIH, Bethesda, ML, USA) software. ICAM-1 expression area ratio was calculated by area of ICAM-1 (pixels of intensity value 10–255) divided by endothelial cell area. In addition, the average fluorescence intensity was calculated from the average of the pixels where fluorescent intensity was more than 9, then normalized by mean of average fluorescent intensity of normal condition. VE-cadherin area ratio was calculated by VE-cadherin area (pixels of intensity value 15–255) divided by the total area (area of endothelial cell + VE-cadherin) according to a previous study [43]. Adhered and trans-endothelial migrated THP-1 cells were counted in entire region of BV channel and migration distance was measured from wall of endothelium to closest pixel of THP-1 cells using ImageJ. Data are expressed by mean ± standard error of mean (S.E.M.) Statistical analysis was conducted by Student’s *t* test, and *p*-value < 0.05 was considered to be statistically significant.

## 3. Results

### 3.1. Simulation of LPS Diffusion and Distribution

**Figure 2:**
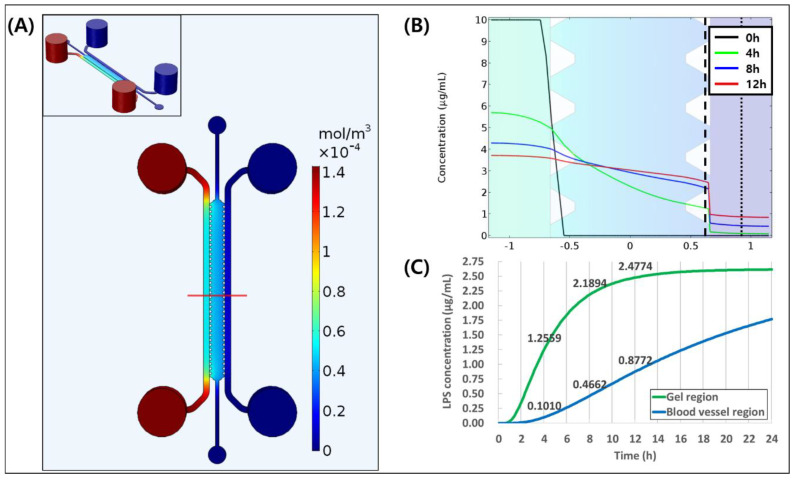
3D simulation of LPS diffusion in microfluidic chip. (**A**) The distribution of Lipopolysaccharide (LPS) concentration inside the channel at 12 h. (**B**) The graph of LPS distribution of red line of (**A**) at 0, 4, 8, and 12 h. (**C**) LPS concentration in gel region (dashed line in (**B**)) and in the blood vessel region (dotted line in (**B**)).

The simulation of diffusion of LPS was conducted in time frame of 0 to 12 h as shown in Figure 2A. LPS concentration distribution of red line in Figure 2A showed that LPS was well diffused on 4 h, which formed almost linear concentration distribution. After 8 and 12 h, LPS concentration distribution was almost flattened (Figure 2B). We also showed the barrier effect of the endothelial layer at the gel–media channel interface, which resulted in concentration drop of the LPS from gel to the blood vessel. In addition, we observed change of LPS concentration over time in gel region (dashed line in Figure 2B) and the blood vessel region (dotted line in Figure 2B) as shown in Figure 2C. LPS concentration of 0.10 μg/mL at 4 h is enough to induce an inflammatory response to blood vessel at the respective time, which was equal or higher than the previous study [44]. Through simulation, we were able to confirm that LPS concentration is linearly increased inside the blood vessel, and we can achieve the concentration that would be high enough to induce inflammation. Therefore, we performed the experiments based on the simulation results.

### 3.2. Intercellular Adhesion Molecule-1 (ICAM-1) Expression on Endothelial Cells

**Figure 3:**
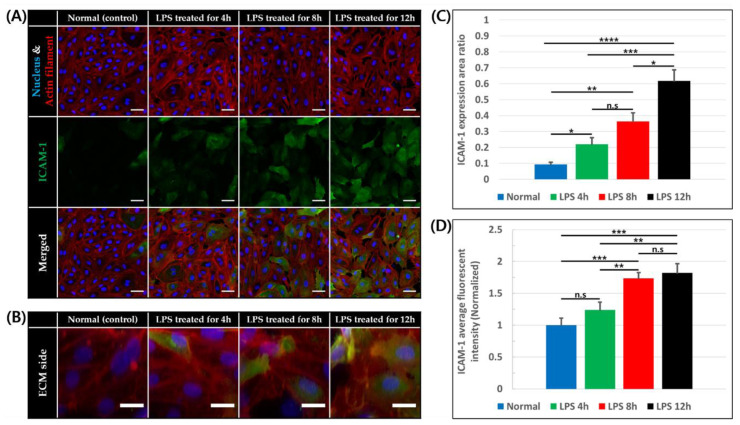
ICAM-1 expression of endothelial cells of 0, 4, 8, and 12 h after LPS treatment. (**A**) Confocal image of endothelial cells on basal plane stained for nucleus (blue), actin filament (red), and ICAM-1 (green), scale bar = 50 μm. (**B**) Z-stack image of endothelial cells on ECM side stained for nucleus (blue), actin filament (red), and ICAM-1 (green), scale bar = 20 μm. (**C**) Ratio of ICAM-1 expression area to endothelial cell area on basal plane (n = 3 × 3, n.s: not significant *: *p* < 0.05, **: *p* < 0.01, ***: *p* < 0.001, ****: *p* < 0.0001) (**D**) Average fluorescent intensity of ICAM-1 on basal plane normalized by mean of normal condition (n = 3 × 3, n.s: not significant, *: *p* < 0.05, **: *p* < 0.01, ***: *p* < 0.001).

Intercellular adhesion molecule-1 (ICAM-1), which is a key adhesion molecule for leukocyte adhesion and trans-endothelial migration, is expressed at a low level on vascular endothelium and can be increased by inflammatory stimuli [45]. For this reason, ICAM-1 expression level of the vessel was observed and quantified in response of application of the inflammatory stimulus. As LPS was diffused into the constructed blood vessels, ICAM-1 expression of endothelial cells at the bottom of the BV channel and on side which is adjacent to the ECM region was shown (Figure 3A,B). Measuring ICAM-1 expression area ratio of basal plane of constructed blood vessel, the ratio was higher with LPS treatment for 4 h (22.04 ± 4.05%, *p* < 0.05), 8 h (36.25 ± 5.51%, *p* < 0.01), 12 h (61.81 ± 6.81%, *p* < 0.0001) than in normal condition (9.31 ± 1.37%) as shown in Figure 3C. In addition, when normalized average fluorescent intensity of ICAM-1 expression of endothelial cells on bottom, the intensity was also higher in 4 h (1.24 ± 0.12), 8 h (1.73 ± 0.09, *p* < 0.001), 12 h (1.82 ± 0.14, *p* < 0.001) than in normal condition (1 ± 0.11) as shown in Figure 3D. On the other hand, ICAM-1 expression of blood vessel was not significantly increased with 150 min of LPS treatment (Figure S2A–C). Overall, the ICAM-1 expressing endothelial cells and intensity of ICAM-1 expression were increased as LPS concentration increases and exposed for a longer time. Some endothelial cells exposed to LPS lost their actin filaments and gaps between cells were also observed. Since LPS treatment for 12 h made integrity of endothelial cells lost considerably, 12 h treatment was excluded in the following experiments.

### 3.3. VE-Cadherin Expression on Endothelial Cells

**Figure 4:**
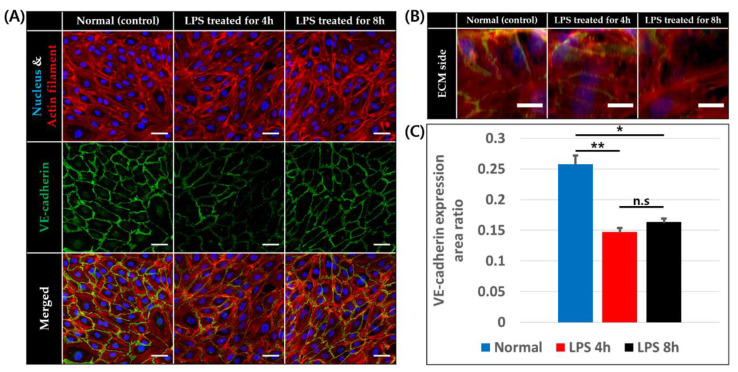
VE-cadherin expression of endothelial cells at 0, 4, and 8 h after LPS treatment. (**A**) Confocal image of endothelial cells on basal plane stained for nucleus (blue), actin filament (red), and VE-cadherin (green), scale bar = 50 μm. (**B**) Z-stack image of endothelial cells on ECM side stained for nucleus (blue), actin filament (red), and VE-cadherin (green), scale bar = 20 μm. (**C**) VE-cadherin expression area to endothelial cell area ratio on basal plane (n = 4 × 3 for normal and LPS treated for 4 h, n = 3 × 3 for LPS treated for 8 h, n.s: not significant *: *p* < 0.0001, **: *p* < 0.00001).

VE-cadherin is a cell–cell adhesion molecule specifically expressed on the endothelial cells, and is downregulated by inflammation [46]. The downregulation of VE-cadherin expression was observed for endothelial cells both on the base plane and ECM side of the BV channel and loss of actin filament and cell free region occurred when LPS was applied for 4 and 8 h, as shown in Figure 4A,B. Furthermore, we measured the VE-cadherin expression area ratio of the blood vessels on bottom part. The ratio was significantly lower with LPS treatment for both 4 h (14.71 ± 0.68%, *p* < 0.00001) and 8 h treatment (16.37 ± 0.57%, *p* < 0.0001) than in normal conditions (25.80 ± 1.39%) as expected. However, the VE-cadherin area ratio of the blood vessels treated by LPS for 4 and 8 h were not significantly different. However, significant downregulation of VE-cadherin was not observed in LPS treatment for 150 min (Figure S2D,E).

### 3.4. THP-1 Cell Adhesion/Migration Assay

**Figure 5:**
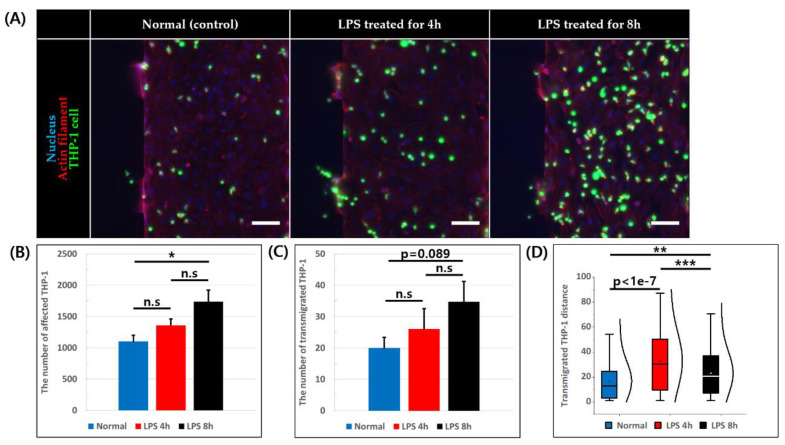
THP-1 adhesion and trans-endothelial migration assay. (**A**) Image of constructed blood vessel and adhered and migrated THP-1 stained for nucleus (blue), actin filament (red), and THP-1 (green), scale bar = 100 μm. (**B**) Number of adhered THP-1 to HUVEC monolayer and migrated THP-1 into collagen. (n = 4, n.s: not significant *: *p* < 0.05) (**C**) Number of transmigrated THP-1 across the endothelial monolayer into collagen. (n = 4, n.s: not significant) (**D**) Distance transmigrated THP-1 travelled measured by the distance between transmigrated THP-1 and HUVEC monolayer. (n = 80 from four devices for normal condition, n = 104 from four devices for LPS 4 h condition, n = 139 from 4 devices for LPS 8 h condition, **: *p* < 0.01, ***: *p* < 0.001).

THP-1 cell suspension was added into the HUVEC coated BV channel, and its adhesion and migration behavior was observed after 6 h of further incubation (Figure 5A). The number of affected THP-1 cells was calculated by summing the number of adhered cells to endothelium and number of cells that have undergone trans-endothelial migration into collagen. The total number of affected THP-1 was 1103.5 ± 95.4, 1360.5 ± 98.5, and 1737.6 ± 183.3 in normal, LPS 4 h, and LPS 8 h condition, respectively, and showed significant difference when LPS was treated for 8 h compared to the normal condition (Figure 5B). Furthermore, we observed the number of THP-1 cells that have undergone trans-endothelial migration and the values were 20 ± 3.4 for normal, 26 ± 6.5 for LPS 4 h, and 34.8 ± 6.4 for LPS 8 h in condition (Figure 5C). Although there seems increasing trend of migrated cells with longer LPS treatment, there were no statistically significant difference. Finally, we have measured the transmigration distance from the endothelium of those THP-1 cells that have undergone trans-endothelial migration, and the average distances were 16.79 ± 1.70 μm (normal), 32.82 ± 2.24 μm (LPS 4 h), and 22.98 ± 1.46 μm (LPS 8 h) with THP-1 cells migrating significantly more in both of the inflamed conditions compared to the normal condition (Figure 5D).

## 4. Discussion

Blood vessel of 3D monolayer was constructed in microfluidic chip, and then the inflammatory response of blood vessel was induced by treating lipopolysaccharide, recapitulating LPS invasion from the basal side of blood vessel. As a result, the ICAM-1 expressions of endothelial cells were gradually increased with the exposed time. ICAM-1 expression is expected to increase even further with the prolonged exposure [47]. This is in line with the results from the previous studies using 2D in vitro model that reported upregulation of ICAM-1 expression of endothelial cells, which increased with longer exposure of LPS [48,49]. We note that although one other study using microfluidic chip reported that ICAM-1 expression was not increased by LPS treatment when only endothelial cell was cultured [30], the difference might be caused by lower LPS concentration and shorter treatment time than our study’s condition. To confirm, we have performed the additional experiments and also showed that both expression of ICAM-1 and VE-cadherin of blood vessels treated by LPS for 150 min was not significantly different from controls (Figure S2). This allow us to conclude that the LPS conditioning time is critical to induce significant inflammation of blood vessel in microfluidic device. As reported in previous studies [50,51], we also observed downregulation of VE-cadherin by inflammatory response to LPS. In result of both ICAM-1 and VE-cadherin experiment, loss of actin filament and gaps between cells were caused by longer LPS treatment time. Thus, these results indicate that inflammation of blood vessel model induced by LPS was well established in our study.

Although different from primary monocyte, THP-1 has been used as immune cell model in numerous studies [31]. According to THP-1 adhesion/migration assay in this study, the number of adhered and trans-endothelial migrated THP-1 tended to increase when LPS exposure time was longer. There was statistically significant difference between normal and LPS 8 h, but no significant difference between Normal and LPS 4 h and between LPS 4 h and LPS 8 h. These results are different from previous studies of in vitro 2D model that THP-1 cells adhered to and transmigrated through HUVECs more in the LPS-treated condition than the normal condition [13]. When monocytic THP-1 cells were co-cultured in blood vessel constructed in microfluidic chip, reduced ZO-1 and occludin expression, and permeability of HUVEC monolayer was observed in previous study [36]. This indicates that co-culturing THP-1 cell can also induce inflammation of endothelial cells. In our experiment, THP-1 cells continuously contact or roll over endothelial cells during seeding until the flow stopped. It is presumed that persistent contact or rolling of THP-1 cells induce endothelial cells to express adhesion protein on endothelial cells for all cases, possibly diminishing the distinct effect of different LPS treatment conditions.

In addition, the number of trans-endothelial migrated THP-1 also had tendency to increase with longer LPS treatment, although not statistically significant. However, more THP-1 cells were trans-endothelially migrated when endothelial cells were exposed to LPS in the previous study [13]. One reason why the number of trans-endothelially migrated THP-1 did not have statistically significant difference with LPS treatment in our experiment may be due to the width to height aspect ratio of constructed vessel, which is about 4:1. Because the width of the channel is longer, THP-1 cells were more likely to be affected by base plane of endothelium than the cells on the ECM side. In addition, the tissues around the blood vessels in vivo are composed of an extracellular matrix as well as stromal cells such as fibroblasts. The fibroblast in connective tissue stimulated by LPS earlier than endothelium can produce pro-inflammatory cytokine such as MCP-1 [52], which can attract THP-1 cells [53] to endothelial cell on ECM. However, when compared the distance of trans-endothelial THP-1 migration, there was a statistically significant difference between conditions. Furthermore, THP-1 cells migrated from endothelium furthest 4 h after exposure to LPS, whereas they were shortest in the controls. This result is quite surprising as it indicates once THP-1 cells contact endothelial cells on ECM side, they were attracted by LPS. As our simulation results show that LPS concentration gradient was formed at 4 h after LPS treatment and flattened by 8 h (Figure 2B), it may be possible that THP-1 cells detected the gradient of LPS, or moved toward the higher LPS concentration.

Using a microfluidic device, we made a 3D monolayer blood vessel exposed to increasing concentration of LPS over time and observed the inflammatory response of endothelial cells and monocytic THP-1, which was difficult to achieve in a 2D monolayer blood vessel. However, our developed model also has number of limitations. First, only endothelial cells were used in our blood vessel model despite a more complex blood vessel structure in vivo, as mentioned above, and in addition, our engineered blood vessel did not include vascular cells that are either organ or disease specific.

## 5. Conclusions

In conclusion, we have developed a microfluidic system that mimics inflammatory response in blood vessels to a lipopolysaccharide that infiltrates blood vessels. For the future direction of this research, an in vivo-like blood vessel consisting of endothelial and stromal cells in ECM could be established in a microfluidic chip, for studying more physiological inflammatory response and the reaction of immune cells. In addition, screening of preclinical anti-inflammatory drug could be conducted in the developed system. Finally, it would be possible to expand our research by investigating a more specific relationship between immune cells and 3D constructed blood vessels.

## Supplementary Materials

The following are available online at https://www.mdpi.com/2072-666X/11/8/747/s1. Figure S1. (A) Dimension of gel channel, media or cell channel, and post in microfluidic chip. (B) Regions of interest, which is about 3.5mm apart (1/4, 2/4, 3/4 point along entire blood vessel channel respectively); Figure S2. Response to LPS treatment for 150 min (2.5 h) of endothelial cells in microfluidic device. (A) Confocal image of endothelial cells on basal plane stained for nucleus (blue), actin filament (red), and ICAM-1 (green) and scale bar = 50 μm. (B) Ratio of ICAM-1 expression area to endothelial cell area (n = 3 × 3, n.s: not significant) (C) Average fluorescent intensity of ICAM-1 normalized by mean of normal condition (n = 3 × 3, n.s: not significant) (D) Confocal image of endothelial cells on basal plane stained for nucleus (blue), actin filament (red), and VE-cadherin (green, bottom), scale bar = 50 μm. (E) VE-cadherin expression area to endothelial cell area ratio (n = 3 × 3, n.s: not significant).
