# Supplementary material for: Lipopolysaccharide-Induced Vascular Inflammation Model on Microfluidic Chip
Source: Micromachines (Basel). 2020 Jul 31;11(8):747. doi: 10.3390/mi11080747 (PMC7465530; doi:10.3390/mi11080747)
Supplement: Supplementary file 1 [file micromachines-11-00747-s001.pdf]

micromachines

Supplementary Materials

Lipopolysaccharide-induced Vascular Inflammation Model on Microfluidic Chip

Ungsig Nam, Seunggyu Kim, Joonha Park and Jessie S. Jeon

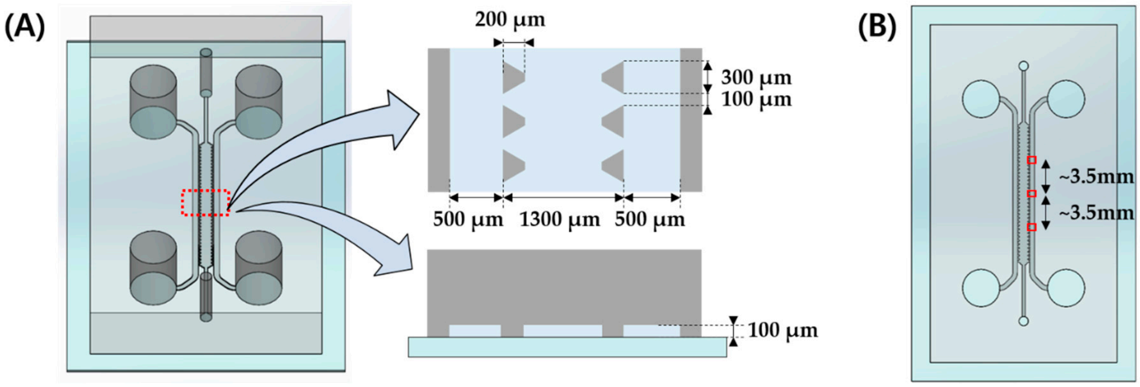

Figure S1. (A) Dimension of gel channel, media or cell channel, and post in microfluidic chip. (B) Regions of interest, which is about 3.5mm apart (1/4, 2/4, 3/4 point along entire blood vessel channel respectively)

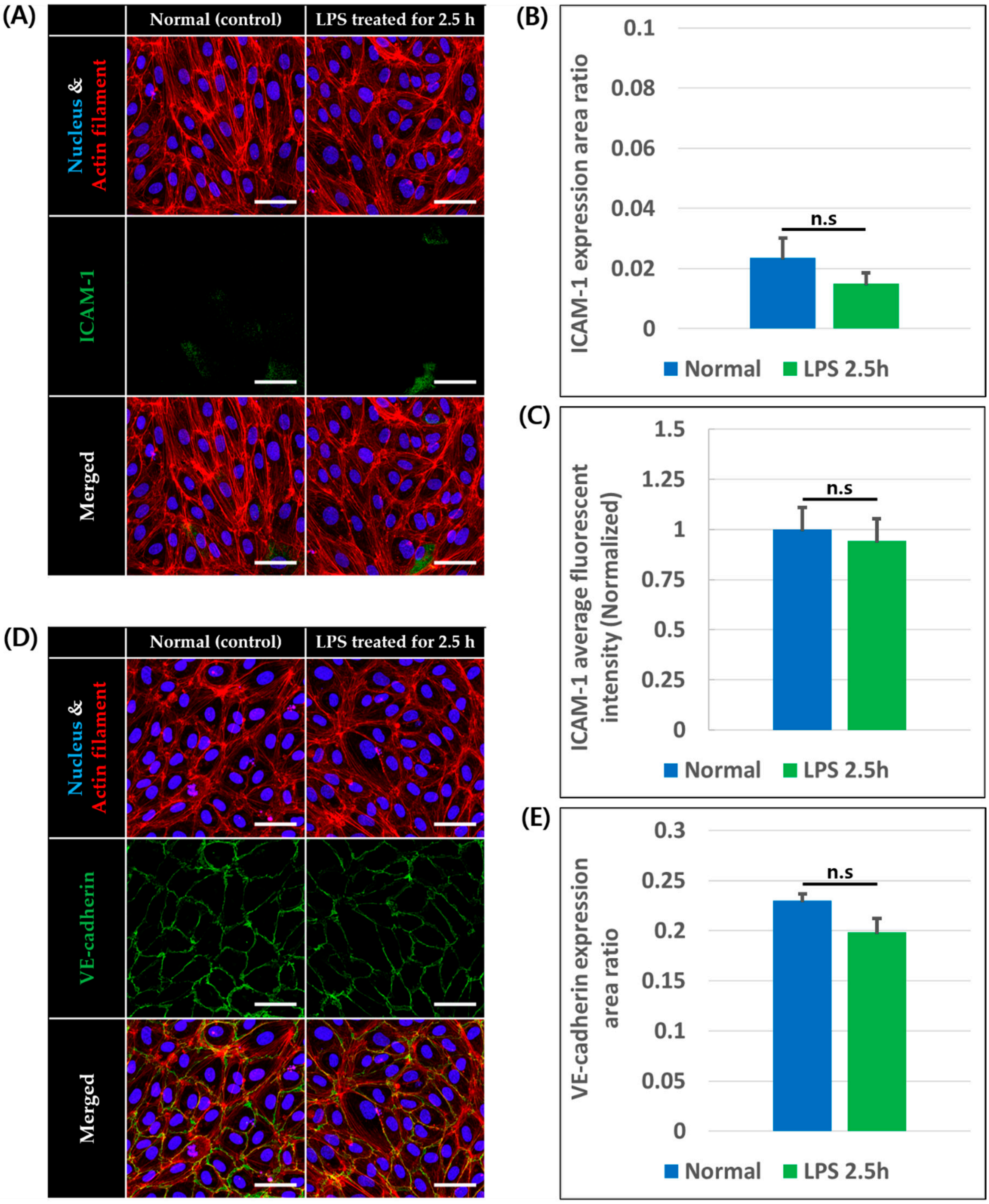

Figure S2. Response to LPS treatment for 150 minutes (2.5 h) of endothelial cells in microfluidic device. (A) Confocal image of endothelial cells on basal plane stained for nucleus (blue), actin filament (red), and ICAM-1 (green) and scale bar=50µm. (B) Ratio of ICAM-1 expression area to endothelial cell area (n=3×3, n.s: not significant) (C) Average fluorescent intensity of ICAM-1 normalized by mean of normal condition (n=3×3, n.s: not significant) (D) Confocal image of endothelial cells on basal plane stained for nucleus (blue), actin filament (red), and VE-cadherin (green, bottom), scale bar=50µm. (E) VE-cadherin expression area to endothelial cell area ratio (n=3×3, n.s: not significant)
